# Production of Two Novel Methoxy-Isoflavones from Biotransformation of 8-Hydroxydaidzein by Recombinant *Escherichia coli* Expressing *O*-Methyltransferase SpOMT2884 from *Streptomyces peucetius*

**DOI:** 10.3390/ijms161126070

**Published:** 2015-11-24

**Authors:** Chien-Min Chiang, Hsiou-Yu Ding, Ya-Ting Tsai, Te-Sheng Chang

**Affiliations:** 1 Department of Biotechnology, Chia Nan University of Pharmacy & Science, No. 60, Sec. 1, Erh-Jen Rd., Jen-Te District, Tainan 71710, Taiwan; cmchiang@mail.cnu.edu.tw; 2 Department of Cosmetics Science, Chia Nan University of Pharmacy & Science, No. 60, Sec. 1, Erh-Jen Rd., Jen-Te District, Tainan 71710, Taiwan; hsiou221@yahoo.com.tw; 3 Department of Biological Sciences and Technology, National University of Tainan, No. 33, Sec. 2, Shu-Lin St., Tainan 70005, Taiwan; s10056024@gm2.nutn.edu.tw

**Keywords:** 7,4′-dihydroxy-8-methoxy-isoflavone, 8,4′-dihydroxy-7-methoxy-isoflavone, 8-hydroxydaidzein, melanogenesis, inhibition, *O*-methyltransferase, SpOMT2884

## Abstract

Biotransformation of 8-hydroxydaidzein by recombinant *Escherichia coli* expressing *O*-methyltransferase (OMT) SpOMT2884 from *Streptomyces peucetius* was investigated. Two metabolites were isolated and identified as 7,4′-dihydroxy-8-methoxy-isoflavone (**1**) and 8,4′-dihydroxy-7-methoxy-isoflavone (**2**), based on mass, 1H-nuclear magnetic resonance (NMR) and 13C-NMR spectrophotometric analysis. The maximum production yields of compound (**1**) and (**2**) in a 5-L fermenter were 9.3 mg/L and 6.0 mg/L, respectively. The two methoxy-isoflavones showed dose-dependent inhibitory effects on melanogenesis in cultured B16 melanoma cells under non-toxic conditions. Among the effects, compound (**1**) decreased melanogenesis to 63.5% of the control at 25 μM. This is the first report on the 8-*O*-methylation activity of OMT toward isoflavones. In addition, the present study also first identified compound (**1**) with potent melanogenesis inhibitory activity.

## 1. Introduction

Isoflavones exist in some plants, such as soybeans [1]. Two major isoflavones found in soybeans are daidzein and genistein, which have been demonstrated to prevent hormone-dependent diseases [2]. In recent years, more studies have focused on *ortho*-hydroxyisoflavones due to their pharmaceutical activities, including anti-cellular proliferation [3], aldose reductase inhibition [4], tyrosinase inhibition [5], anti-mutagenesis [6], anti-melanogenesis [7], and enhancement of cancer chemotherapeutic activity [8]. Due to the numerous bioactivities of *ortho*-hydroxyisoflavones and their rarity in nature, we recently focused on their production. We have successfully produced 8-hydroxydaidzein, 6-hydroxydaidzein, and 3′-hydroxygenistein using *Aspergillus oryzae* and recombinant *Pichia pastoris*, and the maximum production yields of 3′-hydroxygenistein and 6-hydroxydaidzein were the highest in the literature [9,10,11].

*O*-Methylation modification is part of the biosynthesis of some isoflavones and plays a key role in secondary metabolism in plants [12]. Many *O*-methylated isoflavones have been found in various plants, and some studies reported that *O*-methylation of isoflavones would increase biological activities of the isoflavones. For example, formononetin (4′-methoxydaidzein) was shown to have 10-fold anti-melanogenesis activity compared to that of daidzein [13]. In addition, the methylated flavonoids showed higher metabolic stability, membrane permeability, and, thus, greater bioavailability than non-methylated flavonoids [14,15]. Therefore, *O*-methylation is an important strategy for producing specific *O*-methylated isoflavones with specific activities.

In biological systems, *O*-methyltransferase (OMT) catalyzes *O*-methylation through a methyl group donor, *S*-adenosylmethionine (SAM) [12]. OMTs are ubiquitous in nature, and most plant OMTs possess high substrate specificity. In contrast, OMTs from microorganisms contain more flexibility in substrate specificity than those from plants [12]. However, there has been no report on the 8-*O*-methylation activity of OMT toward isoflavones until now, although many OMTs have been studied [12]. Recently, an OMT from *Streptomyces peucetius* ATCC 27952 (SpOMT2884) was reported to catalyze *O*-methylation of 7,8-dihydroxyflavone to produce 7-hydroxy-8-methoxyflavone [16]. Based on the structural similarity between 8-hydroxydaidzein and 7,8-dihydroxyflavone, which both contain 7,8-dihydroxyl-phenyl groups in the flavonoid skeleton, in the present study, we used OMT to catalyze 8-hydroxydaidzein methylation and characterized the chemical structures and bioactivity of the resulting metabolites.

## 2. Results and Discussion

### 2.1. Expression of SpOMT2884 in Escherichia coli

**Figure 1:**
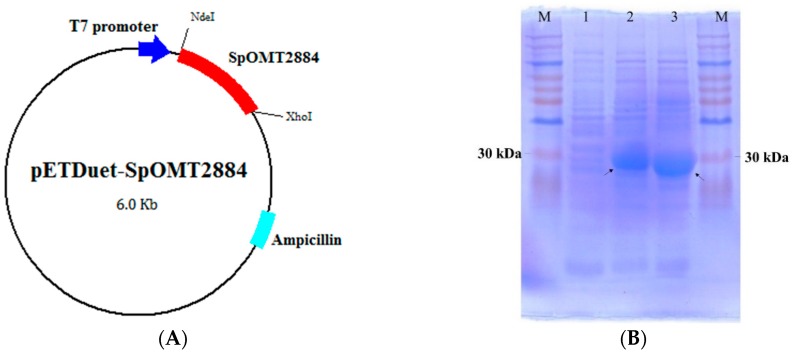
Construction and expression of SpOMT2884 in *E. coli*. (**A**) Map of the expression vector pETDuet-SpOMT2884; (**B**) Sodium dodecyl sulfate polyacrylamide gel electrophoresis (SDS-PAGE) of recombinant *E. coli* harboring pETDuet-SpOMT2884. Crude proteins from whole cell lysis with 0 h induction (lane 1), 4 h induction (lane 2), and 8 h induction (lane 3) were separated with SDS-PAGE. Arrows indicate the expressed SpOMT2884. M represents the molecular weight markers.

To optimize the expression of the *Streptomyces* SpOMT2884 gene in *E. coli*, the gene was chemically synthesized with codon optimization based on the preferences of *E. coli*, and the synthesized SpOMT2884 gene was subcloned into pETDuet-1^TM^ (Novagen, Madison, WI, USA) to form the expression vector pETDuet-SpOMT2884 (Figure 1A). The recombinant *E. coli* harboring the expression vector was confirmed to produce considerable SpOMT2884 protein after isopropyl-β-d-thiogalactopyranoside (IPTG) induction (Figure 1B). Therefore, the recombinant cells were used for the biotransformation study.

### 2.2. Biotransformation of 8-Hydroxydaidzein by the Recombinant E. coli

**Figure 2:**
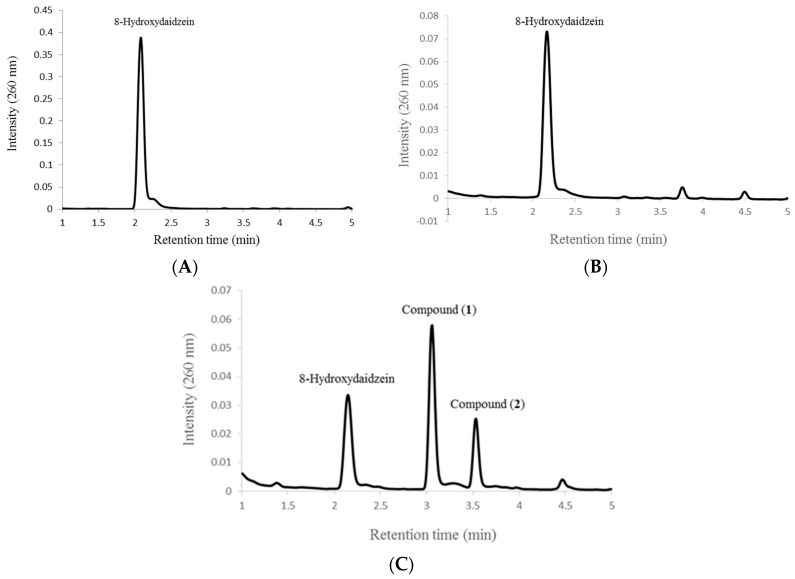
Ultra-performance liquid chromatography (UPLC) profiles of 8-hydroxydaidzein standard (**A**) and fermentation broth ((**B**): 0 h induction; (**C**): 24 h induction) of recombinant *E. coli* expressing SpOMT2884. The recombinant strain was cultivated at 37 °C in shacking flasks with LeMaster and Richards minimal medium (LR medium) containing 100 μM 8-hydroxydaidzein. After induction with 0.5 mM isopropyl-β-d-thiogalactopyranoside (IPTG), aliquot samples of the fermentation were collected and analyzed with UPLC. The detailed protocols for fermentation and UPLC are described in Materials and Methods.

For studying biotransformation, the substrate 8-hydroxydaidzein was added with IPTG in *E. coli* cultivation, and the products’ profiles were determined with ultra-performance liquid chromatography (UPLC) to check whether the substrate could be converted by the recombinant cells. As shown in Figure 2, two major metabolites, compounds (**1**) and (**2**), appeared as new peaks at retention times of 3.2 and 3.7 min, respectively, in the profile of the fermentation broth at 24 h of incubation. The metabolites were further isolated using a preparative high-performance liquid chromatography (HPLC) method and were identified using spectrophotometric methods. Compound (**1**) showed an [M − H]^+^ ion peak at *m*/*z*: 283 in the electrospray ionization mass (ESI-MS) spectrum corresponding to the molecular formula C_16_H_12_O_5_. Then ^1^H and ^13^C nuclear magnetic resonance (NMR) was performed to elucidate the structure. The full assignment of the ^1^H and ^13^C NMR signals was conducted according to heteronuclear multiple quantum coherence (HMQC), heteronuclear multiple bond connectivity (HMBC), and correlation spectroscopy (COSY) spectra. The following data were collected for compound (**1**): ^1^H-NMR (DMSO-*d*_6_, 500 MHz) δ: 8.32 (1H, s, H-2), 7.69 (1H, d, *J* = 8.5 Hz, H-5), 7.36 (2H, d, *J* = 8.5 Hz, H-2′, 6′), 6.99 (1H, d, *J* = 8.5 Hz, H-6), 6.80 (2H, d, *J* = 8.5 Hz, H-3′, 5′), 3.85 (3H, s, OCH_3_); ^13^C-NMR (DMSO-*d*_6_, 125 MHz) δ: 175.2 (C-4, C=O), 157.4 (C-4′), 155.8 (C-7), 153.0 (C-2), 151.0 (C-9), 135.0 (C-8), 130.4 (C-2′, 6′), 123.5 (C-3), 122.7 (C-1′), 121.0 (C-5), 117.2 (C-10), 115.8 (C-6), 115.3 (C-3′, 5′), 61.0 (OCH_3_). The HMBC spectrum revealed a methoxyl proton signal at δ 3.85(s) correlated to carbon resonance at δ 135.0 (C-8). Based on these spectral data and with the comparison of ^1^H-NMR and ^13^C-NMR data in the literature [17], compound (**1**) was characterized as 7,4′-dihydroxy-8-methoxy-isoflavone. Compound (**2**) was obtained as pale yellow powder, showed an [M − H]^+^ ion peak at *m*/*z*: 283, and ^1^H-NMR (DMSO-*d*_6_, 500 MHz) δ: 8.33 (1H, s, H-2), 7.57 (1H, d, *J* = 8.8 Hz, H-5), 7.37 (2H, d, *J* = 8.5 Hz, H-2′, 6′), 7.20 (1H, d, *J* = 8.8 Hz, H-6), 6.80 (2H, d, *J* = 8.5 Hz, H-3′, 5′), 3.91 (3H, s, OCH_3_); ^13^C-NMR (DMSO-*d*_6_, 125 MHz) δ: 175.7 (C-4, C=O), 157.3 (C-4′), 153.4 (C-2), 151.4 (C-7), 146.1 (C-9), 134.9 (C-8), 130.4 (C-2′, 6′), 123.2 (C-3), 122.8 (C-1′), 118.7 (C-10), 115.4 (C-5), 115.3 (C-3′, 5′), 110.5 (C-6), 56.6 (OCH_3_). The methoxyl group of the compound was demonstrated at the C-7 position of the isoflavone by ^1^H-^13^C long-range correlation between H of OCH_3_ and C-7 of isoflavone. By comparing these data with the values in the literature [18], compound (**2**) was identified as 8,4′-dihydroxy-7-methoxy-isoflavone.

**Figure 3:**
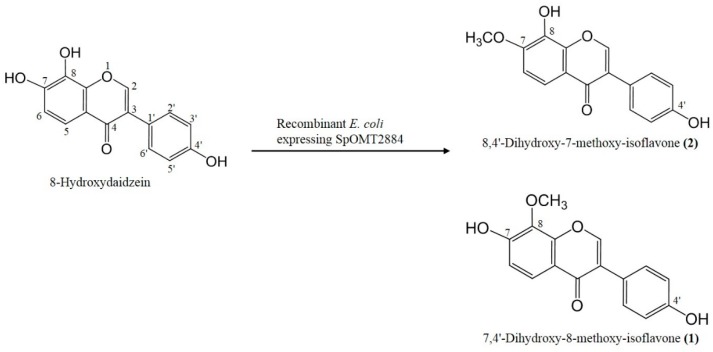
Diagram of the biotransformation of 8-hydroxydaidzein by the recombinant *E. coli* expressing SpOMT2884.

Based on the resolved structures of the two metabolites, we suggested the biotransformation process of 8-hydroxydaidzein by the recombinant *E. coli* as shown in Figure 3. Because we did not observe any other major peaks during the biotransformation, OMT activity on 4′-*O*-methylation of 8-hydroxydaidzein does not seem to occur. In addition, none of the double methylation product was found, which indicated that SpOMT2884 could catalyze only 7-*O*-methylation or 8-*O*-methylation. Koirala *et al.* found that SpOMT2884 catalyzed 8-*O*-methylation of 7,8-dihydroxyflavone (7,8-DHF) to form 7-hydroxy-8-methoxy-flavone but did not observe 7-*O*-methylation activity on either 7,8-DHF or on 7-hydroxy-8-methoxy-flavone [16]. They also found that 7-hydroxy-4′-methoxy-isoflavone (formononetin) was a substrate of SpOMT2884. Taking the results together, although 7,8-DHF and 8-hydroxydaidzein share the same structural part of the dihydroxy-phenyl group, SpOMT2884 catalyzes either 7-*O*-methylation or 8-*O*-methylation in 8-hydroxydaidzein. The enzyme catalyzes only 8-*O*-methylation of 7,8-DHF. This reaction preference requires additional enzymatic structural studies.

**Figure 4:**
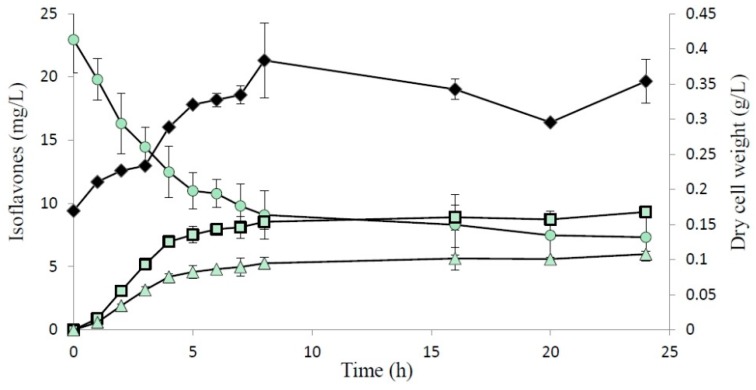
Cell growth (filled symbols) and isoflavone (open symbols) profiles of the recombinant *E. coli* in a 5 L fermenter. The 8-Hydroxydaidzein and compounds (**1**) and (**2**) are represented by the circle, square, and triangle symbols, respectively. The average (*n* = 2) is presented with the standard deviation represented by error bars.

The production profile of the two methoxy-isoflavones in a 5 L fermenter is shown in Figure 4. The maximum production yields of compounds (**1**) and (**2**) in the biotransformation were 9.3 and 6.0 mg/L, respectively. In the results, the products accumulated rapidly within the initial 6 h after induction and then at a very slow rate. However, the substrate and the expressed SpOMT2884 were still of a significant amount after 6 h of induction (Figure 1B and Figure 4). The possible reason for the decreased enzymatic conversion rate after 6 h of induction is the insufficiency of cofactor SAM. It has been reported that co-expression of the SAM synthetase gene could increase OMT activity [19]. Additional studies should be conducted to evaluate the effect of the co-expression of the SAM synthetase gene on the biotransformation of 8-hydroxydaidzein by recombinant *E. coli*.

**Figure 5:**
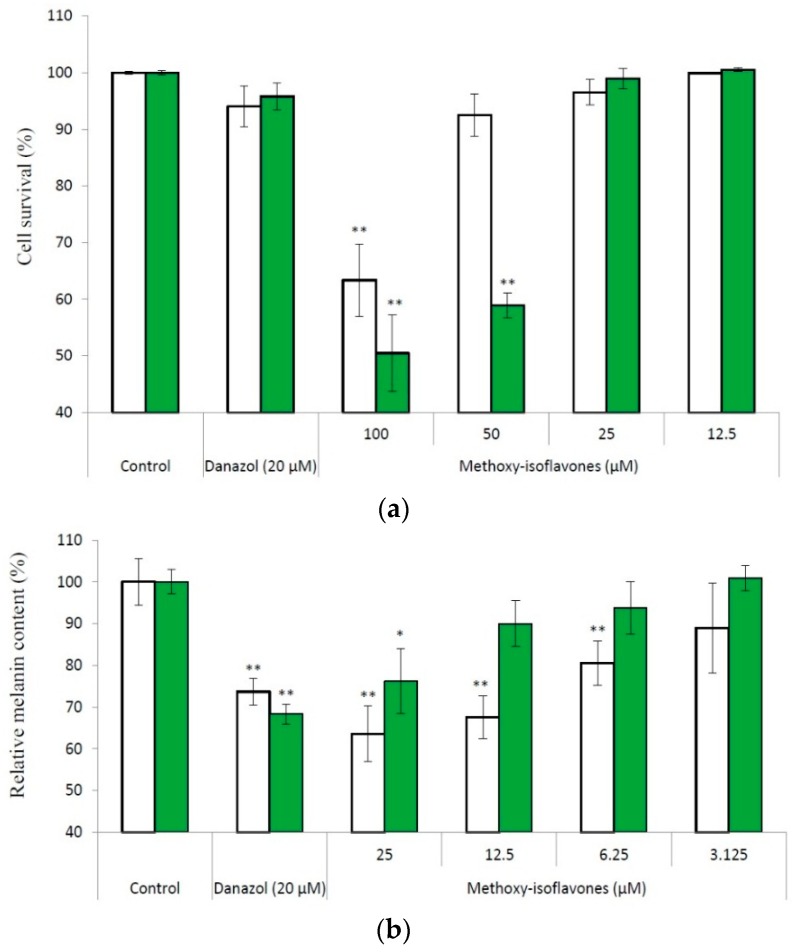
Effects of compounds (**1**) (open bars) and (**2**) (filled bars) on the cell survival (**a**) and melanogenesis (**b**) of B16 cells. The cells were incubated for 1 day, and then treated with drugs for another 2 days. Following cultivation, the cell viability and cell melanin content were measured with the protocols described in Materials and Methods. The average (*n* = 3) is shown, with the standard deviation represented by error bars. The data were compared with those of the control using the Student's *t*-test, and *p* < 0.01 (*); *p* < 0.001 (**) was considered to be statistically significant.

### 2.3 Inhibitory Activity on Melanogenesis of B16 Cells

Methylation of flavonoids could increase the chemical stability, membrane permeability, and then the bioactivity of flavonoids. We found that the methyl derivatives of daidzein and genistein (formononetin and biochanin A) possess potent melanogenesis inhibitory activity [13]. Therefore, we investigated the inhibitory effects of the two methoxy-isoflavones on melanogenesis in cultured B16 melanoma cells. We first determined the ranges of non-toxic concentrations of the compounds toward the B16 cells with the 3-(4,5-dimethylthiazol-2-yl)-2,5-diphenyltetrazolium bromide (MTT) method. The results showed that both methoxy-isoflavones did not exhibit cytotoxicity under a concentration of 25 μM (Figure 5A). Accordingly, we used 25 μM as the maximum concentration in the determination of the inhibition of melanogenesis, and the result showed that both methoxy-isoflavones dose-dependently inhibited melanogenesis of B16 cells in non-toxic concentrations (Figure 5B). At 25 μM of compound (**1**), the melanin content of the treated cells remained at 63.5% of the control. Moreover, the inhibition by compound (**1**) was greater than that produced by danazol, which has previously been identified as a potent melanogenesis inhibitor [20].

Compound (**1**) was first purified from *Abrus mollis* in 2006 [21] and then from *Zornia diphylla* [17] and from *Spatholobus suberectus* [22] in 2012. Compound (**2**) was isolated from *Maackia amurensis* in 2001 only [18]. Due to the rarity of these two methoxy-isoflavones in nature, there have been few reports on the bioactivity of these isoflavones. The present study first identified compound (**1**) with potent melanogenesis inhibitory activity. In addition, the present study holds promise in a mass production method for the two isoflavones by use of recombinant *E. coli* if yields can be increased through further process improvements. This opens up pharmaceutical research and applications of the two methoxy-isoflavones in the future.

## 3. Materials and Methods

### 3.1. Microorganisms, Cells and Chemicals

We purchased both mouse B16 melanoma cells (4A5) and *A. oryzae* BCRC 32288 from the Bioresources Collection and Research Center (BCRC, Food Industry Research and Development Institute, Hsinchu, Taiwan). The expression system containing both *E. coli* BL21 (DE3) and vector pETDuet-1^TM^ was obtained from Novagen. IPTG, MTT, and 3-isobutyl-1-methylxanthine (IBMX) were purchased from Sigma (St. Louis, MO, USA). The 8-Hydroxydaidzein was isolated from 10 L fermentation broth of *A. oryzae* BCRC 32288 with 150 mg/L of daidzein feeding in yeast peptone dextrose medium according to our previous report [9]. The other reagents and solvents used were of high-quality and were purchased from commercially available sources.

### 3.2. Expression of SpOMT2884 in E. coli

The *Streptomyces* SpOMT2884 gene (GenBank protein database accession number KF420279) was chemically synthesized with codon optimization based on the preferences of *E. coli* by GenScript (Piscataway, NJ, USA). The synthesized SpOMT2884 gene was subcloned into the pETDuet-1™ vector through the NdeI and XhoI sites to obtain the expression vector pETDuet-SpOMT2884. The expression vector was transformed into *E. coli* BL21 (DE3) via electroporation to obtain the recombinant *E. coli* used for the biotransformation.

### 3.3. Fermentation and UPLC

The recombinant *E. coli* harboring expression vector was cultivated in 20 mL of LR medium containing 50 μg/mL of ampicillin and 0.4% glycerol, with 200 rpm shaking at 37 °C [23]. As the optical density at 600 nm reached 0.6, 0.5 mM of IPTG and 0.1 mM of 8-hydroxydaidzein were added to induce expression of SpOMT2884 and start the biotransformation. At indicated time intervals, 0.5 mL of cultures was extracted with 0.5 mL of MeOH/CAN (50%:50%) and analyzed with UPLC, which was operated through an analytic C18 reversed-phase column (Acquity UPLC BEH C18, 1.7 μm, 2.1 i.d. × 100 mm, Waters, Milford, CT, USA) and a gradient elution using water (A) containing 1% (*v*/*v*) acetic acid and methanol (B) with a linear gradient for 5 min with 15% to 35% B at a flow rate of 0.3 mL/min, injection volume of 0.2 μL, and detection of the absorbance at 260 nm [11].

### 3.4. Scale-up Fermentation, Isolation and Identification of Biotransformation Products

A seed culture of recombinant *E. coli* (100 mL) was inoculated into a 5 L fermenter containing 2.5 L LR medium supplemented with 0.4% glycerol, followed by cultivation with aeration (0.5 vvm) and agitation (280 rpm) at 37 °C. After induction with 0.5 mM of IPTG, the fermentation was continued for another 24 h. A 10 mL aliquot of the culture was collected at several different time intervals and analyzed with UPLC. The purification process was the same as in our previous work [11] and is described briefly below. Two batches of 2.5 L fermentation broth were prepared for the purification of the biotransformation products. Following fermentation, total broth was twice extracted with an equal volume of ethyl acetate, and the extracts were combined and condensed under a vacuum. The residue was then suspended in 200 mL of 50% methanol. After filtration with a 2.2 μm nylon membrane under a vacuum, the metabolite was purified by preparative HPLC methods. The operational conditions for the preparative HPLC analysis by a preparative C18 reversed-phase column (Inertsil, 10 μm, 20.0 i.d. × 250 mm, ODS 3, GL Sciences, Eindhoven, The Netherlands) included a gradient elution using water (A) and methanol (B) with a linear gradient for 25 min with 25% to 50% B at a flow rate of 15 mL/min, and detection of the absorbance at 260 nm. The injection volume was 10 mL. The elution corresponding to the peak of the metabolite in the UPLC analysis was collected, condensed under a vacuum, and then crystallized by freeze-drying. Finally, 17.5 mg of compound (**1**) and 16.0 mg of compound (**2**) were obtained, and the structures of the compounds were confirmed with NMR and mass spectral analysis.

### 3.5. Determination of Cell Viability and Melanin Content

Determinations of cell viability and melanin content were performed as previously reported [24] and described briefly below. Dulbecco’s modified Eagle’s medium (DMEM) containing 10% (*v*/*v*) fetal bovine serum was used to cultivate the B16 cells, which were incubated at 37 °C in a humidified, CO_2_-controlled (5%) incubator. After 1 day of incubation, the cells were treated with tested drugs in the presence of a melanogenesis stimulation agent (400 μM of IBMX) for another 2 days. Then, the drug-treated cells were harvested and the cell viability and melanin content were measured according to our previous study [24].
